# Apolipoprotein E Gene Variation in Pakistani Subjects with Type 2 Diabetes with and without Cardiovascular Complications

**DOI:** 10.3390/medicina60060961

**Published:** 2024-06-10

**Authors:** Shehwar Nadeem, Tahir Maqbool, Javed Anver Qureshi, Awais Altaf, Sadia Naz, Muzammal Mateen Azhar, Inam Ullah, Tawaf Ali Shah, Muhammad Usman Qamar, Ahmad Mohammad Salamatullah

**Affiliations:** 1 Centre for Research in Molecular Medicine, Institute of Molecular Biology and Biotechnology, The University of Lahore, Lahore 54660, Pakistan; dr.shehwar.nadeem@live.com (S.N.); javed.anver@imbb.uol.edu.pk (J.A.Q.); awaisaltaf362@yahoo.com (A.A.); inamullah@imbb.uol.edu.pk (I.U.); 2 Department of Allied Health Sciences, The University of Lahore, Lahore 54660, Pakistan; sadiaumair902@gmail.com (S.N.); mateen0092009@gmail.com (M.M.A.); 3 College of Agriculture Engineering and Food Sciences, Shandong University of Technology, Zibo 255049, China; tawafbiotech@yahoo.com; 4 Division of Infectious Disease, Department of Medicine, University of Geneva, 1211 Geneva, Switzerland; muhammed.qamar@etu.unige.ch; 5 Institute of Microbiology, Faculty of Life Sciences, Government College University Faisalabad, Faisalabad 38000, Pakistan; 6 Department of Food Science and Nutrition, College of Food and Agriculture Sciences, King Saud University, Riyadh 11451, Saudi Arabia; asalamh@ksu.edu.sa

**Keywords:** apolipoprotein E, polymorphisms, ischemic heart disease, stroke

## Abstract

*Background:* Apolipoprotein E (APOE) gene polymorphism has been implicated in the pathogenesis of various metabolic disorders, including type 2 diabetes mellitus (T2DM). Type 2 diabetes mellitus (T2DM) is a major public health concern worldwide, including in Pakistan. Cardiovascular problems linked with T2DM have a significant impact on individuals and society. The goal of this study is to investigate the relationship between Apolipoprotein E (ApoE) genotypes, dyslipidemia, and cardiovascular complications such as ischemic heart disease (IHD) and stroke. *Methods:* This study was carried out on 260 subjects divided into controls and diabetics. The diabetics were further divided into four subgroups such as D1: diabetics without cardiovascular issues, D2: diabetics with heart disease, D3: diabetics with stroke, and D4: diabetics with both heart disease and stroke. Anthropometric parameters (age, BMI) and risk factors (smoking, diabetes duration, hypertension) were assessed in all groups. Serum levels of TC, TG, LDL, HDL, VLDL, creatinine, BSF, and HbA1c were also measured. Apolipoprotein E gene polymorphism was determined using PCR-RFLP. *Results:* Hypertension, BMI, and dyslipidemia are defined as elevated levels of total cholesterol, triglycerides, LDL, and VLDL, and decreased levels of HDL. Uncontrolled hyperglycemia (elevated fasting blood sugar and glycated hemoglobin) in T2DM was linked to vascular complications such as IHD and stroke. Hypertension was prevalent in 79.3% of the population. Stage 2 hypertension was more prevalent in all age groups. It was also noted that common genotypes in the Pakistani population are 3/3, 4/4, 2/3, and 3/4. The frequency of genotypes 3/4 and 2/3 is highest in diabetics with stroke. Genotype 3/3 is present frequently in diabetics with IHD/stroke and patients with both these complications. However, genotype 4/4 is most frequently found in diabetics with IHD. *Conclusions:* It is concluded that BMI, hypertension, hyperglycemia, atherosclerosis, and dyslipidemia are linked with cardiovascular complications of type 2 diabetes. Apolipoprotein E gene polymorphism is associated with cardiovascular disease in patients with diabetes by affecting the lipid profile.

## 1. Introduction

Diabetes is a chronic metabolic disease characterized by high blood glucose levels that result from defects in the body’s ability to produce or utilize insulin. Diabetes mellitus has a complex pathogenesis [1]. A raised blood sugar level is the main biomarker for establishing the diagnosis of diabetes. Diabetes is diagnosed at random blood glucose greater than or equal to 200 mg/dL, fasting blood sugar levels more than 126 mg/dL, and HbA1c (glycated hemoglobin that shows a record of previous three months’ blood sugar) greater than 6.5%. Diabetes is an epidemic that exists worldwide. It is non-communicable and a threat to both affluent and non-affluent people. According to the International Diabetes Federation, the prevalence of diabetes varies in different countries. About 537 million adults in the world were diabetic in the year 2021. The number is expected to rise to 700 million by 2045. In the same year, 26.7% of adults in Pakistan were affected by diabetes and the total number of cases was approximately 33 million. Medical experts believe that lack of exercise, dietary habits, and rising obesity are contributing to the surge in diabetes in Pakistan.

Lee et al., 2022 [2], indicated that diabetes can lead to microvascular damage (retinopathy, nephropathy, and neuropathy) and macrovascular complications (ischemic heart disease, stroke, and peripheral vascular disease). It has been reported that various demographic, clinical, and biochemical parameters such as age, gender, socioeconomic status, duration of diabetes, BMI, hypertension, BSF, HbA1c, lipid profile, creatinine, and genetic factors are also responsible for cardiovascular complications like IHD and stroke in these patients [3].

The APOE gene is a multifunctional 34-Kda glycoprotein containing 299 amino acids, situated on the long arm of chromosome 19 at q13.2. It is a multiform gene with single-nucleotide polymorphisms (SNPs) at 112 and 158 positions, resulting in three important alleles: ε2, ε3, and ε4. It has three isoforms: APOE2 (Cys112/Cys158), APOE3 (Cys112/Arg158), and APOE4 (Arg112/Arg158). APOE3 is the most frequently found gene. APOE isoforms have variable effects on lipoprotein metabolism. Apolipoproteins are proteins that bind to lipids (such as cholesterol and triglycerides) to form lipoprotein particles, which are essential for transporting lipids through the bloodstream. These proteins play crucial roles in lipid metabolism. It is mostly synthesized in the liver and a very small quantity is produced in the small intestine. Apolipoprotein has various subfamilies, A, B, C, D, E, L, F, H, M, N, and R, for different functions. Each person inherits two APOE alleles, one from each parent, which means that people can have six different combinations of genotypes. Genotypes of APOE are E2/E2, E2/E3, E3/E3, E3/E4, E4/E4, and E2/E4. In previous studies, it was found that genetic factors also lead to cardiovascular complications in type 2 diabetics. Apolipoprotein E is a highly important gene that is linked with cardiovascular complications (ischemic heart disease and stroke) due to dyslipidemia and atherosclerosis in type 2 diabetes. Dyslipidemia or lipoprotein abnormalities can speed up diabetic vascular complications and can cause atherosclerotic changes in these subjects [4]. APOE allele ε4 might cause risk for both T2DM and CVD independently [5]. Apolipoprotein E gene polymorphism is also responsible for diabetic macrovascular complications.

Genotypes E4/4 and 3/4 were found to be associated with an increased risk of cardiovascular diseases in type 2 diabetics among men and women. However, genotype 2/2 is cardioprotective and individuals carrying this genotype have less chance of developing cardiac problems. Patients with diabetes who carried E3/E4 or E4/E4 genotypes had higher concentrations of serum TC, TG, LDL-C, and VLDL, and lower levels of HDL (protective cholesterol) than patients with E2/E2 or E2/E3 genotypes.

Complicated molecular pathways that involve raised blood sugar levels and resistance to insulin actions in addition to hypertension, obesity, and dyslipidemia defined by high levels of total cholesterol, triglycerides, LDL, and VLDL with low levels of HDL (good cholesterol) lead to cardiovascular complications in individuals with diabetes [6,7,8].

An increased body mass index (BMI > 25 kg/m^2^) is thought to be an important factor in diabetics. In the past few years, it has been reported that the coexistence of diabetes and hypertension exacerbates cardiovascular complications in individuals with diabetes and is a significant risk for vascular complications related to type 2 diabetes [9]. It was also observed that the frequency of hypertension is twice in patients with diabetes as compared to non-diabetics and these patients have insulin resistance. Previously, Ghosh et al., 2017 [10], observed a strong link between obesity with insulin resistance and atherosclerosis leading to diabetic vascular complications.

In subjects with diabetes with no past history of myocardial infarction (MI), the 7-year risk of developing MI is greater in diabetics as compared to non-diabetics. However, in patients with a past history of myocardial infarction, the risk of redeveloping MI is much greater in diabetics in seven years [11]. It indicates that diabetes has a significant contribution to the development of MI. However, a study was performed on adults 30 years or older in Denmark. It was reported that diabetes increases the risk of coronary heart disease [12].

Cardiovascular disease (CVD) is the most frequent cause of mortality in individuals with diabetes. About 52% of mortality in T2DM is due to cardiovascular complications. Many reports indicate a strong association between diabetes and cardiovascular episodes [13]. Very recently, it has been noticed that even a prediabetic state with impaired fasting glycemia (IFG) or impaired glucose tolerance (IGT) is linked with morbidity and mortality due to cardiovascular disease [14]. 

In addition to chronic heart disease (CHD), there is an increased risk of developing stroke in diabetes because of damaged vessels of the body and stroke is an emergency. Diabetics have about twice the chance of developing stroke as compared to non-diabetic subjects, particularly in the case of ischemic stroke. Hyperglycemia is linked with a poor prognosis of stroke [9]. The role of Apolipoprotein E gene polymorphism has not been observed in Pakistani diabetics with regard to demographic, clinical, and biochemical parameters.

Objectives of the present study: To evaluate the association of Apolipoprotein gene polymorphism with ischemic heart disease and stroke in type 2 diabetics.

## 2. Materials and Methods

### 2.1. Study Design 

Study Design: This is a “retrospective study”. Both disease cases and controls were selected from the Social Security Hospital, Lahore. Study subjects were segregated into the control (C) and the diabetic groups (D1–D4), Group 1, D1 (diabetics without any accompanying comorbidities) (DM only); Group 2, D2 (diabetics with ischemic heart disease) (DM + IHD); Group 3, D3 (diabetics with stroke) (DM + STROKE); and Group 4, D4 (diabetics with ischemic heart disease and stroke) (DM + IHD + STROKE) [15,16]. 

#### Inclusion Criteria for Healthy Individuals and Individuals with Diabetes

Inclusion and Exclusion Criteria: Both inclusion and exclusion criteria were carefully taken into consideration while selecting the controls and subjects with disease for the present study. The selection of the study subjects was based on medical reports, family history, and physical examination. All the study subjects (both genders) were of age 40 and above [16]. Controls were healthy adults accompanying the patients with diabetes. They had no diabetes (normal HbA1c less than 6.5%), and no hypertension (normal blood pressure “BP” less than 120/80 mm/Hg, no ischemic heart disease, and no stroke). Patients with type 2 diabetes with and without complications of diseases like ischemic heart disease (IHD) and stroke (CVA) as evident from medical records were selected for this study. Pregnant women, lactating women, patients with malignant tumors, and patients with renal disease/advanced renal disease and familial hyperlipidemia were excluded from this study.

### 2.2. Patients with Renal Disease

#### 2.2.1. Collection of Demographic and Clinical Data

Demographic data were gathered from the selected subjects whereas the examination was performed by the concerned medical professional in a clinical setting. All the subjects were recruited from the Department of Medicine, Social Security Hospital, Lahore. It also included patients admitted to the ward as well as those visiting the outpatient department regularly.

#### 2.2.2. Sample Collection

**Figure 1:**
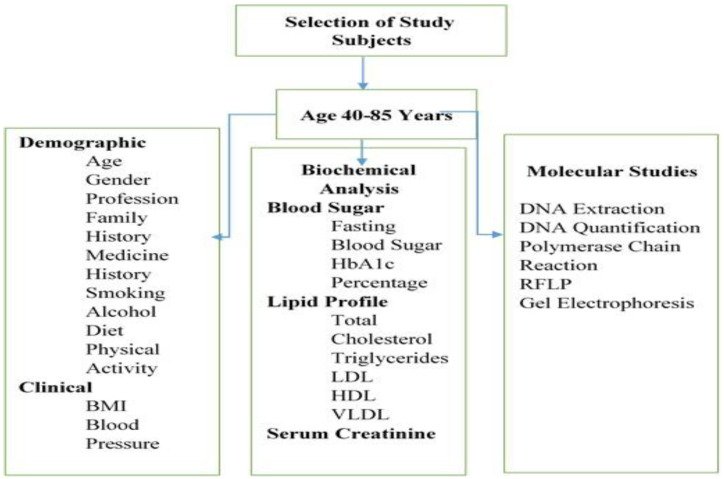
Experimental plan showing demographic, clinical, biochemical, and genetic analysis.

Venus blood samples were taken aseptically from all study subjects. For this, a ten (10)-milliliter blood sample was collected through venipuncture and distributed equally in two (02) customized blood collecting tubes, with and without anti-coagulants such as ethylenediamine tetra acetic acid (EDTA). These samples were transported under cold conditions to the laboratory at the Institute of Molecular Biology and Biotechnology (IMBB), The University of Lahore. The tubes containing the coagulated blood samples were centrifuged at 5000 rpm for 10–15 min (centrifuge Sigma 3-30k, Burladingen, Germany). Supernatant plasma was pipetted out and stored at −20 °C for later use, whereas uncoagulated blood was used for DNA extraction. The schematic diagram of the experimental plan is presented in Figure 1.

Anthropometric measurements are noninvasive quantitative measurements of the body. In this regard, the core elements of anthropometry, i.e., height and body weight, were taken into account to calculate the body mass index (BMI). Weight and height of the subjects were obtained from the study subjects and BMI was calculated according to the standard formula [BMI = weight (kg)/[height (m)]^2^ described by Bhupathiraju et al., 2016 [17]. Blood pressure assessment of the study subjects was measured by a sphygmomanometer as described earlier [18,19].

### 2.3. Biochemical Estimations

All the biochemical estimations were carried out according to Mansoor et al., 2022. Biochemical estimations were performed on a Beckman coulter AU 480 Chemistry Analyzer (Brea, CA, USA). Fasting blood sugar levels in the study subjects were measured by using the commercial Glucose Oxidase method known as GLU-OX available from DIRUI, China, by the spectrophotometric method. The glycated hemoglobin (HbA1c) level was estimated by the Latex agglutination method using commercial kit GLYCOHEMOGLOBIN A 1 c), DIRUI, Changchun, China. Fasting lipid profiles such as total cholesterol (TC), triglycerides (TGs), and high-density lipoprotein cholesterol (HDL-C) levels were estimated in the study samples using commercially available kits from DIRUI, China. Low-density lipoprotein was calculated by using the modified Friedewald formula (LDL-C (mg/dL) = Non-HDL-C × 90% minus TG × 10) described by Chen et al., 2010 [20]. Very low-density lipoprotein (VLDL) levels were estimated by using the Friedewald equation described as VLDL = Triglycerides/5 by Oliveira et al., 2013 [21]. Creatinine was measured by an enzymatic method using a commercially available kit (CRE-ENZYME), DIRUI, China.

### 2.4. DNA Extraction and Quantification

DNA was extracted by a blood genomic DNA extraction kit (QIAAMP DNA mini kit, Qiagen, Batch no. 166030545, Briogene Pvt. Ltd., Hilden, Germany) according to the manufacturer’s guidelines. In total, 20 µL of this mixture served as a DNA source for amplification. DNA concentration was measured by the Nanodrop [22]. In some cases, DNA concentration was measured by ultraviolet absorbance spectrometry at 260 nm. This wavelength corresponds to 50 µg of double-stranded DNA per mL. The ratio of absorbencies at 260 nm and 280 nm was taken as 1.8 with a pure sample. A ratio of less than 1.8 indicates that the preparation is contaminated with phenol or protein.

#### 2.4.1. Polymerase Chain Reaction (PCR) Amplification

Conventional PCR was performed according to Maqbool et al., 2019, with 100 ng of purified genomic DNA, 0.5 µM of APOE P1 and APOE P2 primers, P1 forward sense 5′-GGCACGGCTGTCCAAGGA-3′, P2 forward sense 5′-CCCACCTGCGCAAGCTGCGC-3′ and P3 Reverse Antisense 5′-CTCGCGGATGGCGCTGAG-3′. The reaction mixture contained 200 µM of a dNTP mixture, 1 M betaine, 2 mm MgCl_2_, 2.5 U Taq DNA polymerase (BioQuest), and a reaction buffer in a 50 µL reaction volume. Each PCR reaction mixture comprised 100 ng (almost 2 µL) of DNA, 0.8 µL of both primers (10 pmol/µL each), an 8 µL master mix, and 5 µL of distilled water. The following steps were performed: the amplification was initiated at 95 °C for 2 min, followed by 45 cycles of 95 °C for 30 s, 68 °C for 30 s, and 72 °C for 30 s, and then a final extension at 72 °C for 7 min.

#### 2.4.2. Restriction Fragment Length Polymorphism (RFLP)

The resulting PCR products (20 µL) were treated with 5 U Hha1, a restriction enzyme, for 3 h. The restricted fragments were separated on 12.5% discontinuous polyacrylamide gel electrophoresis [23] and 3 percent agarose gel.

### 2.5. Statistical Analysis

Data were analyzed by using SPSS version 26.0. Data were described as the mean ± standard deviation (SD) or frequency and percentages as applicable. A one-way analysis of variance was used within the group and two-way analysis of variance (ANOVA) was used between the groups. A *p*-value ≤ 0.05 was considered significant.

## 3. Results

In the present study, subjects with type 2 diabetes from the Lahore region were investigated with or without ischemic heart disease or stroke in connection to their demographic, anthropometric, and biochemical indices or parameters in regards to Apolipoprotein E gene polymorphism. The results are presented under the following headings:

### 3.1. Analysis of Demographic and Clinical Data

Demographic and clinical information of the study subjects is summarized in Table 1.

### 3.2. Characteristics of the Study Groups

Table 2 illustrates the basic demographic and clinical characteristics of the study group subjects, indicating the number of samples, age, BMI, systolic blood pressure, and diastolic blood pressure in each group. All the groups consisted of 52 subjects, who were further divided into males and females, out of which 18 subjects were males and 34 subjects were females. According to results, there were no significant differences in age of both genders in control and diabetic groups and in between groups where *p* = 0.7622

Age: No significant difference in age was found in both genders in control and diabetic groups and in between groups where *p* = 0.7622 (Table 3).

**Figure 2:**
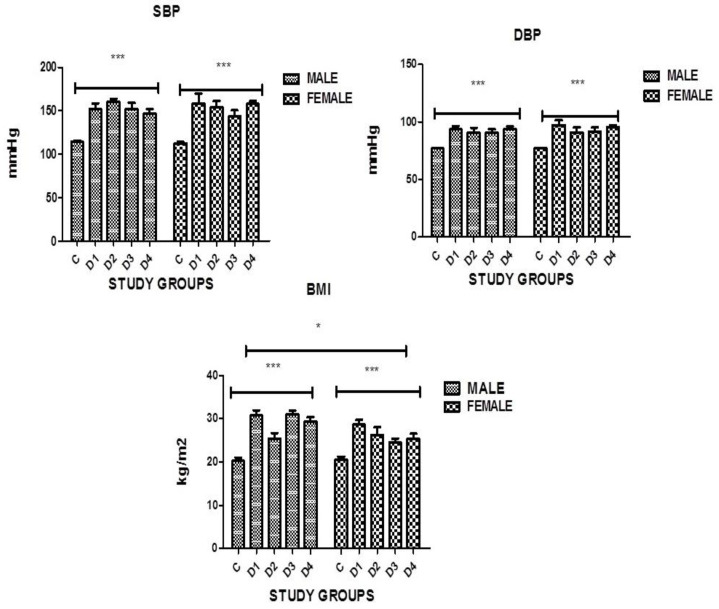
Graphical presentation of BMI, SBP, and DBP in study groups where *p* ≤ 0.05. C: CONTROL; D1: DM ONLY; D2: DM+IHD; D3: DM + STROKE; D4: DM+STROKE+IHD; BMI: BODY MASS INDEX. ERROR BARS are SE. *** represents *p* = 0.001, * represents *p* < 0.05.

Body Mass Index (BMI): The present study data (Table 3, Figure 2) show that BMI was higher in subjects with diabetes and a significant difference was found in both genders in all groups as compared to controls, having the *p*-value 0.0001, which is statistically significant.

Hypertension (B.P): The present study data (Table 3, Figure 2) show that both systolic and diastolic blood pressure was found to be high in subjects with diabetes. A significant difference was found in both genders in all groups as compared to controls, having the *p*-value 0.0001, which is statistically significant.

Table 4 illustrates the stages of hypertension. The data indicated that 79.3% of subjects (25% male and 54.2% female) were hypertensive. The study subjects were assessed for the hypertension stage. A total of 22.5% of subjects (6.2% males and 16.3% females) had stage 1 hypertension. The number of subjects having stage 2 hypertension was 56.6% (18.7% males and 37.9% females). About 20.6% (9.6% males and 11% females) of subjects were normotensive. The prevalence of stage 2 hypertension is more as compared to stage 1.

### 3.3. Effect of Age on Hypertension

It has been documented in the literature that age affects blood pressure, which is an important risk factor for developing cardiovascular complications in diabetics. In the present study, the blood pressure was estimated to define the stages of hypertension and the subjects were divided into two groups according to age (Table 5). Among subjects between 40 and 60 years of age, a total of 41.8% of subjects were hypertensive. A total of 4.8% had stage 1 hypertension, 37.0% of subjects had stage 2 hypertension, and 2.8% were normotensive. Among subjects above 60 years of age, a total of 37.4% of subjects were hypertensive, 8.6 had stage 1 hypertension, and 28.8% had stage 2 hypertension and 17.5% were normotensive. In both groups, most patients had stage 2 hypertension.

### 3.4. Effect of Duration of Diabetes on Hypertension

It has been normally observed that the duration from the onset of diabetes affects blood pressure. The present study indicates that among subjects with a duration of diabetes below 10 years, 54.4% were hypertensive (5.4% stage 1 and 49% stage 2) and among subjects with a duration of diabetes above 10 years, 24.9% were hypertensive (4.9% stage 1 and 20% stage 2). The number of patients with hypertension was more when the duration of diabetes was below 10 years and stage 2 hypertension was more prevalent as compared to stage 1 above and below 10 years (Table 6).

### 3.5. Onset of Diabetes in Study Subjects

Subjects with a duration of diabetes above 10 years made up 21.1% (9.6% males and 11.5% females) and subjects with a duration below 10 years made up 78.8% (25% males and 53.8% females). This shows that there were more subjects with a duration of diabetes less than 10 years in our study (Table 7).

### 3.6. Smoking

As evident by previous studies, smoking increases the risk of cardiovascular complications in subjects with diabetes. Therefore, the present study also focused on assessing the role of demographic and clinical variables in diabetic smokers and non-smokers (Table 8). Smokers and non-smokers in all disease groups had a significantly higher body mass index and systolic and diastolic blood pressure as compared to healthy controls.

### 3.7. Biochemical Analysis

In order to see how the biochemical variables like BSF, HbA1c, fasting lipid profile, and creatinine present in the groups affect the diabetic cardiovascular complications, the distribution of biochemical markers was evaluated in study subjects and groups. The results obtained are presented in Table 9, Table 10, Table 11 and Table 12. In the disease (diabetic) group, the fasting blood sugar (BSF) and glycated hemoglobin (HbA1c) was high. The lipid profile involves total cholesterol (TC), triglycerides (TG), low density (LDL), very low density lipoproteins (VLDL) and high density lipoproteins (HDL). Biochemical variables were significantly raised in all groups when compared with controls except HDL which was found to be decreased. However, in group D1 (DM + IHD), the percentage of HbA1c was noted to be higher in females as compared to males of the same group; however, total cholesterol and triglyceride levels were higher in males of this group. In the present study, it was also observed that the levels of total cholesterol and LDL were higher in males than the female subjects in group D4 (DM + IHD + STROKE). The significance of biochemical data is applicable in the form of p-values; therefore, it was important to calculate the mean ± SEM of the biochemical variables in study groups as presented in Table 11.

### 3.8. Blood Sugar Levels

**Figure 3:**
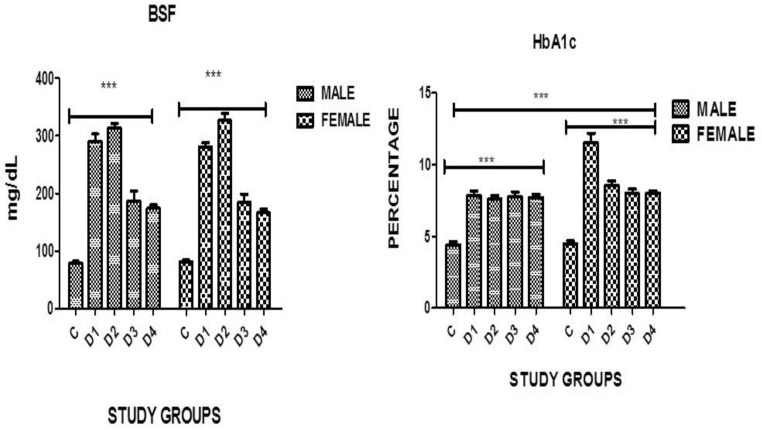
Graphical presentation of BSF and HbA1c in study groups where *p* ≤ 0.05. C: CONTROL; D1: DM ONLY; D2: DM + IHD; D3: DM + STROKE; D4: DM + STROKE + IHD; HbA1c: GLYCATED HEMOGLOBIN; ERROR BARS are SE; *** represents *p* = 0.001.

A highly significant difference in fasting blood sugar was found in both genders in all diseased groups when compared with the controls where *p* = 0.0001. However, no significance was noticed in between the groups, although in diabetics with an IHD level of BSF was higher in females as compared to males of the same group (Table 11, Figure 3).

### 3.9. HbA1c

A highly significant difference in HbA1c was found in both genders in all disease groups when compared with the controls where *p* = 0.0001. A significance was also noticed in the diabetic group without complications where the females had much higher levels as compared to males of the same category (Table 11, Figure 3).

### 3.10. Lipid Profile: Total Cholesterol

**Figure 4:**
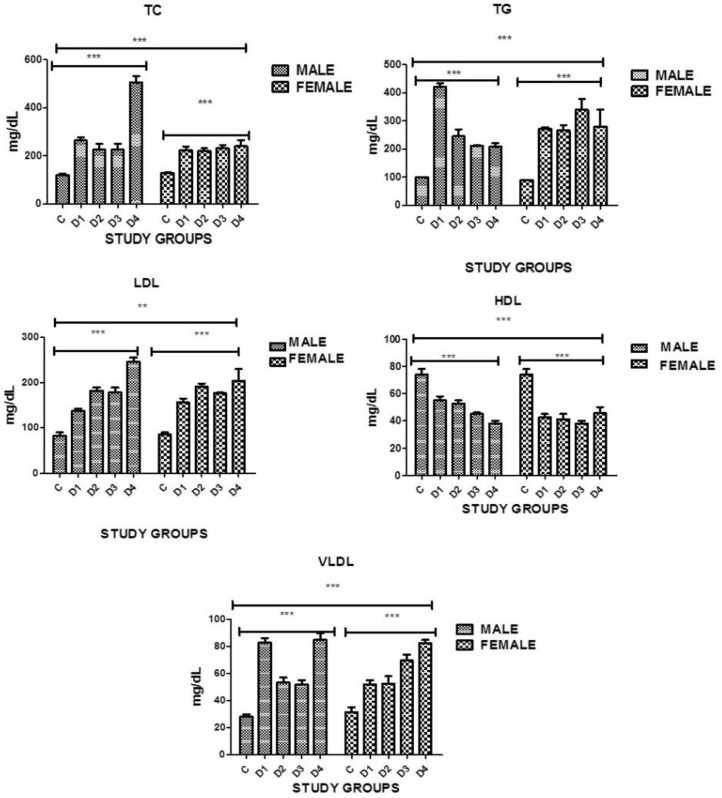
Graphical presentation of TC, TG, LDL, HDL, and VLDL in study groups where *p* ≤ 0.05. C: CONTROL; D1: DM ONLY; D2: DM + IHD; D3: DM+STROKE; D4: DM + STROKE + IHD; HbA1c: GLYCATED HEMOGLOBIN; ERROR BARS are SE; *** represents *p* = 0.001, ** represents *p* = 0.05.

The present study indicated a significant difference in blood cholesterol levels that was found in both genders in all disease groups when compared with the controls. Serum cholesterol was high in all disease groups. However, a significance was noticed in between the groups as well. Among diabetics with stroke and IHD and among diabetics with no complications, males had higher levels of cholesterol as compared to females where *p* = 0.0001 (Table 11, Figure 4).

### 3.11. Total Triglycerides

A highly significant difference in triglyceride levels was found in both genders in all disease groups when compared with the controls. Among diabetics with stroke and IHD and diabetics with stroke, females had higher levels of triglycerides as compared to males. Among diabetics with no complications, the levels of triglycerides were high in males as compared to females, where *p* = 0.0001. However, significance was also noticed in between the groups as well (Table 11, Figure 4).

### 3.12. Low-Density Lipoproteins

A highly significant difference in low-density lipoproteins was found in both genders in all disease groups when compared with the controls. High levels of LDL proteins were seen in all disease groups as compared to controls. Among diabetics with IHD and stroke, levels were high in males as compared to females. Among diabetics with IHD, females showed higher levels of LDL protein as compared to males, where *p* = 0.0001. However, a significance was noticed in between the groups as well (Table 11, Figure 4).

### 3.13. High-Density Lipoproteins

Groups were compared with the controls, where *p* = 0.0001 (Table 11, Figure 4).

### 3.14. Very-Low-Density Lipoproteins

Significantly increased levels of VLDL were seen in both genders and in all disease groups when compared with the controls, where *p* < 0.0001. However, a significance was also noticed in between the groups. Diabetic males with no complications had higher levels of VLDL as compared to females and diabetic females with stroke had higher levels as compared to males of the same group (Table 11, Figure 4).

### 3.15. Serum Creatinine

**Figure 5:**
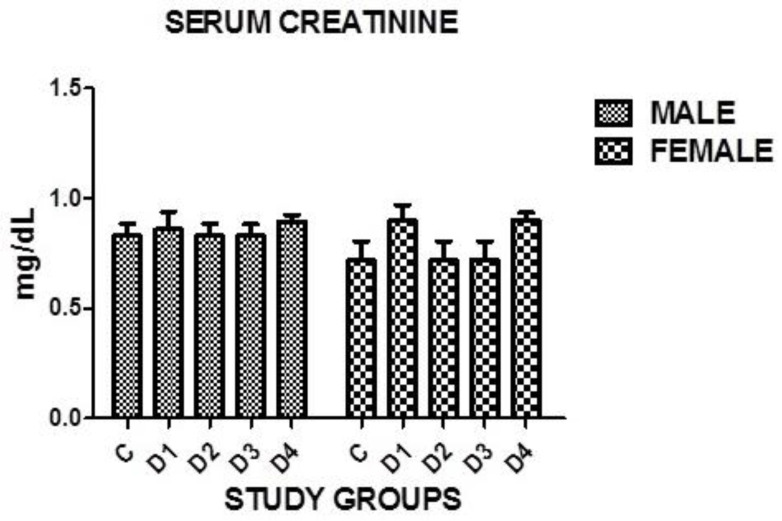
Graphical presentation of serum creatinine in study groups where *p* = 0.1614.

An insignificant difference in serum creatinine was found in both genders in all disease groups when compared with the controls where *p* = 0.1614. However, no significance was noticed in between the groups (Table 11, Figure 5).

### 3.16. Evaluation of Biochemical Parameters in Smokers and Non-Smokers in Study Groups

There is evidence from the present research that smoking can contribute to development of IHD and stroke in subjects with diabetes. Biochemical markers like BSF, HbA1c, total cholesterol, triglycerides, LDL, and VLDL in these individuals were noticed to be significantly higher as compared to healthy controls and HDL was found to be decrease (Table 12).

### 3.17. Restriction Analysis and Genotyping of Amplified DNA Product

**Figure 6:**
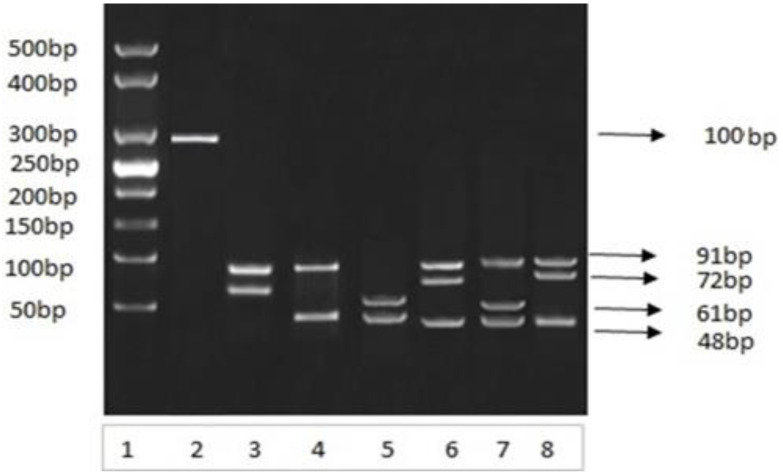
Analysis of PCR-amplified 292 bp product. Restriction analysis of Apolipoprotein E gene on 03 percent agarose gel under electrical field Lane 1 involves molecular-weight DNA marker obtained from study samples. Lane 2 is amplified PCR product; Lanes 3, 4, 5, 6, 7, and 8 contain restricted fragments by using Hha1 restriction enzyme. Restricted fragments were visualized by ethidium bromide staining.

The amplified 292 bp PCR products amplified from different blood samples were subjected to HhaI restriction endonuclease digestion. HhaI restriction endonuclease cleaved the amplified DNA on specific sites, which resulted in a specific restriction pattern for each genotype (Figure 6). The restricted DNA bands were compared with a 25 bp DNA marker and the different individual genotypes were separated and categorized based on the band size criteria.

### 3.18. Genotypic Distribution/Variation

**Figure 7:**
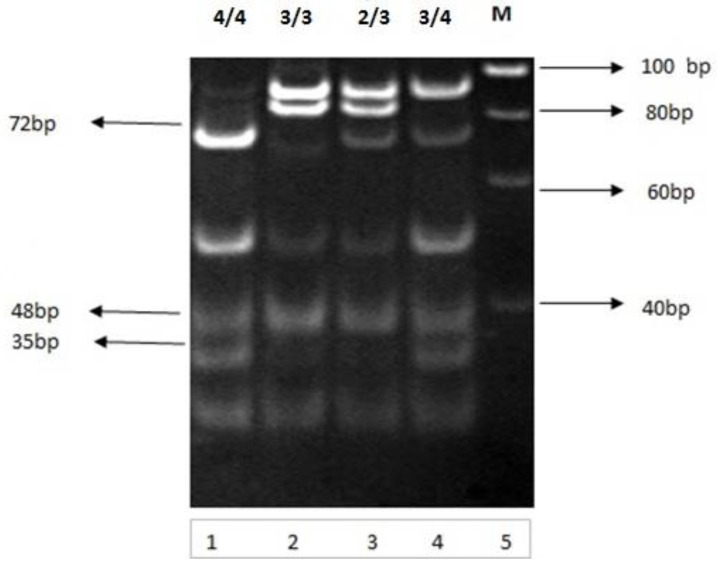
Analysis of PCR-amplified 292 bp product. Restriction analysis and genotyping of Apolipoprotein E gene on 10 percent nondenaturing polyacrylamide gel electrophoresis. Lanes 1, 2, 3, and 4 contain restricted fragments (genotypes 3/3, 4/4, 2/3, and 3/4, respectively) by using Hha1 restriction enzyme. Lane 5 is molecular-weight DNA obtained from study samples. Restricted fragments were visualized by ethidium bromide staining.

The purified and amplified genomic DNA was amplified by the primer pair. The 292 bp PCR products were subjected to HhaI restriction endonuclease digestion. HhaI restriction endonuclease cleaved specific sites, resulting in a specific restriction pattern for each genotype (Figure 7, Table 13). Apolipoprotein E genotypes were determined by analyzing the restricted fragments obtained from restriction (PCR-RFLP). Fragments were separated on nondenaturing polyacrylamide gel electrophoresis. The restricted DNA fragments obtained from PCR-RFLP were 3/3 (91, 61, 48 and 35 bp), E4/4 (72, 61, 48, and 35 bp), E2/3 (91, 83, 61, 48, and 35 bp), and E 3/4 (91, 72, 61, 48, and 35 bp). Figure shows the complete restriction pattern for each genotype in blood samples tested from studied groups.

The genotypes of APOE in the Pakistani type 2 diabetic population were 3/3, 4/4, 2/3, and 3/4. In the study population, the predominant genotype was E3/3, where the E3/3 genotype accounted for 3.8% in the control group (C), 19.2% in diabetics with no complications (D1), 26.9% in the IHD group (D2), 23.0% in diabetics with stroke (D3), and 26.9% in the IHD–stroke group (D4). In the present study, the E3/4 genotype accounted for 5.7% in the control group (C), 15.3% in diabetics without complications (D1), 21.1% in diabetics with IHD (D2), 25% in diabetics with stroke (D3), and 23% in the IHD–stroke group (D4). The frequency of the E 2/3 genotype was increased (23%) in diabetics with ischemic stroke (D3) compared to 5.7% in controls, 11.5% in diabetics with no complications (D1), 15.3% in diabetics with IHD (D2), and 19.2% in the IHD–stroke group (D4). Genotype 4/4 was most frequently found (30.7%) in diabetics with IHD (D2) as compared to controls (3.8%). However, its frequency was 13.4% in diabetics with no complications (D1), 19.2% in diabetics with stroke (D3), and 25% in the IHD–stroke group (D4). In patients with IHD and stroke (D4), the levels of triglycerides, LDL, total cholesterol, and VLDL were increased and HDL levels were found to be significantly decreased.

## 4. Discussion

Diabetes is a metabolic disorder that causes a heavy burden on healthcare systems worldwide. The present study analyzed a representative sample of people from Lahore, Pakistan, and its surrounding regions to investigate the association of demographic, clinical, and biochemical parameters and Apolipoprotein E gene polymorphism with ischemic heart disease and stroke in type 2 diabetics. In the present study, a total of 260 subjects participated. These study subjects were placed equally into five groups. Two groups consisted of controls (healthy controls and type 2 diabetics with no complications), whereas the other three groups were type 2 diabetics with IHD, type 2 diabetics with stroke, and type 2 diabetics with both IHD and stroke. Research by the American Diabetes Association (ADA) showed that management of diabetes is directly affected by gender and age, where females and adults of 60 years and above are affected due to the coexistence of multiple medical conditions involving the heart and vessels, leading to a limitation and insufficiencies of medical prescription. Following the agenda, subjects were divided into females and males.

According to the present retrospective study, the age of the participants was from 40 to 85 years. This age group was selected because previous studies indicated that this age group has a greater chance of becoming hypertensive and diabetic. Moreover, these two diseases are very well known for increasing the danger of cardiac and vascular disease (CVD) and are considered known causes of death globally [22]. Hence, older subjects with type 2 diabetes > 40 years of age were selected to estimate the threat of cardiovascular complications in these subjects.

The present study found that females were more affected by type 2 diabetes and its complications compared to males. Machado et al., 2013, stated that females are highly affected by type 2 diabetes because they are less muscular, which does not support high uptake of fixed glucose load, and have relatively high levels of estrogen and progesterone, which are involved in the reduction in the whole-body insulin sensitivity [24,25,26].

Likewise, it was found that females suffering from cardiovascular complications of diabetes were more numerous than male subjects in all the disease groups (Table 1). Similar findings were reported by others [9,27]. These findings are consistent with a previous analysis in which a 46% increased danger of fatal IHD in women with diabetes was reported by Huxley et al., 2006 [28].

A study by Bradley et al., 2016 [29], suggested that BMI is one of the factors that increases the incidence of diabetes in almost all countries and has a strong connection with insulin resistance [30]. BMI is usually defined as an excessive amount of body fat and is assessed by kg/m^2^. It is a significant causative factor of uncontrolled glucose levels in the blood and diabetic cardiovascular complications [17]. In the present study, 92.7% of subjects (28.3% males and 64.4% females) were obese (Table 1). A significant difference in BMI belonging to both genders was observed in all groups as compared to healthy controls.

Hypertension is very frequently seen in patients with diabetes and is an important risk factor for diabetic cardiovascular complications. In the present study, 79.3% of individuals (24.9% males, 54.2% females) were hypertensive, B.P. > 140/90 (Table 1). In the age group 40–60 years, 41.8% of subjects were hypertensive, 4.8% had stage 1, and 37.0% had stage 2 hypertension. In subjects above 60 years of age, a total of 37.4% of subjects were hypertensive. In total, 8.6% had stage 1 and 28.8% of subjects had stage 2 hypertension, Stage 2 hypertension was frequently present in both age groups (Table 4 and Table 5). A highly significant difference in SBP and DBP was found in both genders in all disease groups. Similar results were reported by Abdelbagi et al., 2021 [31] (Table 1, Table 2, Table 3 and Table 4). Another study reported that the incidence of hypertension in diabetics was high and males had a greater risk of developing hypertension compared to females [32].

Smoking is considered to speed up the risk of cardiovascular complications twofold in type 2 diabetes [33]. In the present study, 11.5% of subjects (all males) gave a history of smoking almost 10 cigarettes/day. Smokers and non-smokers in all disease groups had significantly higher BSF, HbA1c, total cholesterol, triglycerides, LDL, and VLDL and low levels of HDL as compared to healthy controls. Among diabetics with IHD and stroke, the smokers had high levels of total cholesterol and among diabetics with stroke, non-smokers had high levels of triglycerides as compared to smokers (Table 9, Table 10, Table 11 and Table 12). Out of 30 smokers, only 1 was normotensive and others were hypertensive. There were 14 smokers among diabetics with IHD, 5 among diabetics with stroke, 7 among diabetics with both stroke and IHD, and 4 among diabetics with no complications; we can say that the maximum number (26) of smokers belonged to the groups with diabetic complications and 4 belonged to the group of subjects with diabetes without complications. These results regarding smoking are supported by Campagna et al., 2019 [34]; The United Kingdom Prospective Diabetes Study (UKPD); and the Nurses’ Health Study, which reported smoking as an important risk for stroke and CHD in T2DM. Similar results were reported by a Swedish study, in which the relative risk (RR) of smoking was higher for myocardial infarction as compared to stroke in 30- to 59-year-old subjects [35]. Similar observations were also reported in a study carried out by Gavin et al., 2010 [36].

Physical activity can decrease the occurrence of cardiac disease and stroke in type 2 diabetics by improving ischemia, blood sugar levels, and insulin sensitivity as observed by Nilsson et al., 2019 [37]. Exercise is considered to be a significant factor in managing diabetes mellitus. In the present study, only 12.0% of subjects were practicing healthy routines and physical activity 2.3 times/week. Almost 88% of subjects were physically inactive (Table 1). The results of the present study are consistent with observations of Abushamat et al., 2019 [38]. In their study, they observed that 40.8% of individuals with diabetes were physically inactive and were exercising less than 10 min per week. This shows that the majority of people in our society do not give importance to exercise. In a recent meta-analysis by Shah et al., 2021 [39], it was proved by almost all the studies that there is a significant role of exercise in decreasing HbA1c to the point at which the threat of diabetic complications is almost minimal. This is also similar to our results.

Patients with a positive family history of diabetes and cardiovascular disease have the same risks involved. It increases the danger of developing diabetes three times as observed by Tsenkova et al., 2016 [40]. In the current study, family history was positive in only 10.4% of subjects (3.8% males and 3.3% females) (Table 1). However, in a study carried out in Lahore, Pakistan, 64% of individuals gave a positive family history of T2DM. Family history was positive in 67% of subjects among individuals with uncontrolled blood sugar levels and among patients with controlled blood sugar levels, only 55% had positive family history. Data from research carried out on 137 subjects with type 2 diabetes in 2010 reported that diabetics have more significant (*p* = 0.0001) family history of diabetes when compared with non-diabetics [41].

Hyperglycemia and hyperinsulinemia result in increasing amounts of circulating FFA in plasma and atherosclerosis, leading to cardiovascular complications as reported by Wang et al., 2016 [11]. In the present study, about 87% of subjects in the disease groups had uncontrolled fasting blood sugar (>126 mg/dL) and only 13% had controlled fasting blood sugar (<126 mg/dL) (Figure 5). A highly significant difference in fasting blood sugar was found in both genders in all disease groups when compared with controls, where *p* < 0.0001. This is consistent with a study conducted by Riaz et al., 2021 [42]. According to their observation, the majority of subjects (76.9%) had raised fasting blood sugar levels and diabetes was not controlled. Only 24% of subjects had controlled sugar levels. The results of the present study are comparable to a study carried out in Mirpur Khas by Shaikh et al., 2008 [43], which showed that 76% of patients had uncontrolled diabetes. The majority of patients (92%) included in a study who claimed that they were on regular medication had high glucose levels [41]. This is comparable to our results.

Dyslipidemia was also present in 95.5% of the Pakistani population in the current study when compared with controls. Some studies have also reported increased danger of stroke and heart disease in diabetics due to dyslipidemia, especially with elevated levels of LDL-C [44]. LDL-C increases the levels of FFA in plasma after decreased insulin sensitivity develops. The situation becomes more complicated because of high levels of inflammatory cytokines as reported by Narindrarangkura et al., 2019 [45]. In the current study, elevated LDL levels were observed (130 mg/dL) as compared to controls (Figure 4). A highly significant difference in low-density lipoproteins was found in both genders in all disease groups when compared with the controls, where *p* < 0.0001. It was also noticed that 81% of individuals in the disease groups with cardiovascular complications were suffering from mixed dyslipidemia. Cholesterol levels were also found to be high (>200 mg/dL) in both genders as compared to healthy controls and a significance was observed when cholesterol levels were compared with controls in both genders and in all disease groups where *p* < 0.0001. High levels (>150 mg/dL) of triglycerides were observed in both genders as compared to healthy controls. These observations are similar to the study (61% females and 20% males) carried out by Mehta et al., 2021 [46], in Eastern Nepal and Das et al., 2020 [47]. In the present study, a deranged lipid profile due to atherosclerosis was found in the majority of the patients, which could be due to decreased insulin sensitivity enhanced by hyperglycemia and toxicity due to lipid accumulation.

Genetic and epigenetic factors also influence DM, hypertension, obesity, and metabolic syndrome. The genotypes of the APOE gene in the Pakistani type 2 diabetic population were E3/3, E4/4, E2/3, and E3/4. The frequency of genotype E3/3 was high in disease groups compared to controls. Our results are consistent with previous studies carried out by Lebedy et al., 2016 [48], in the Egyptian population. Similarly, the frequency of E3/E4 and 4/4 was significantly higher in diabetics with cardiovascular complications when compared to controls. This is similar to the results obtained by Lebedy et al., 2016 [48].

APOE gene polymorphism was reported in type 2 diabetics in the presence or absence of cardiovascular disease in the Hakka population of southern China. The genotype 3/4 was a significant and strong foreteller of CVD in individuals suffering from T2DM. A significant association between genotype E3/E4 and cardiovascular complications in type 2 diabetics was reported just like previous studies [5]. The most abundantly found genotype was 3/3 in the study. In patients with CVD and T2DM, its frequency increased significantly. Compared with controls, the frequency of E3/E3 was at *p* < 0.01. However, genotype E3/E4 was remarkably increased (*p* < 0.05). This is comparable to our results.

We found only four genotypes in the Pakistani population, i.e., E 3/3, E4/4, E2/3, and E3/4. A study carried out by Liu et al., 2019 [5], showed that APOE gene polymorphism is associated with T2DM and CVD in the Chinese population and E3/4 was significantly elevated in these patients while E3/3 was significantly decreased as compared to controls. Similarly, dyslipidemia was more prevalent in patients with genotype E 4/4 and lower in patients with genotype E2/2. The present study, however, showed a different result. The percentage of both E3/3 and 3/4 was significantly raised in patients with diabetes with cardiovascular complications compared to controls. In fact, the frequency was found to be higher in patients with diabetes without any cardiovascular complications as well when compared with controls. Dyslipidemia was associated with all genotypes. Lebedy et al., 2016 [48], found that subjects with diabetes having cardiovascular complications with genotype ¾ showed higher levels of TC, non-HDL, and LDL. However, no correlation of APOE was found with HDL levels. In the present study, we found that all genotypes, not only E3/4, showed elevated levels of TC, TG, LDL, and VLDL. The levels of HDL were decreased significantly in all genotypes, confirming its association with APOE polymorphism. A study carried out by Ashiq et al., 2021 [49], showed that E4/4 is the reason for cardiac disease in diabetics while allele 2 is cardioprotective, which is different from the present study results. E2/3 and 4/4 were both found to be associated with IHD in addition to stroke in subjects with diabetes. The results of previous studies are unclear and controversial regarding ischemic stroke and the association of the E2/3 genotype in type 2 diabetics. The present study reported an association of E 2/3 with stroke and IHD. In fact, E3/4 and 2/3 were most frequently found in subjects with diabetes with stroke as compared to controls.

The analysis of Ashiq et al., 2021 [49], reported that the presence of allele 4 raised the danger of T2DM by 1.64-fold and the risk of cardiovascular complications by 1.80-fold. In addition, type 2 diabetics with allele 4 had 3.75 times greater chances of developing CVD when the comparison was performed with controls (*p* < 0.01). The research reported that the age group and inhaling cigarettes could also act as dangers of T2DM and CVD independently (*p* < 0.01 and *p* < 0.01, respectively). In this study, lipid levels in individuals with type 2 diabetes having CVD were analyzed. In these subjects, lipid levels were compared between subjects with genotypes E3/E3 and E4/4. It was noticed that TC, TG, and LDL levels in patients with CVD with allele 4 were raised as compared to patients with CVD with the E3/E3 genotype (*p* < 0.05). Levels of HDL in individuals with T2DM and CVD and the ε4 allele were much less as compared to individuals with T2DM and cardiovascular complications and the E3/E3 genotype (*p* < 0.05). However, in the present study, dyslipidemia was equally present in all genotypes; however, the percentage of genotypes varied in different study groups.

Many previous studies have shown that dyslipidemia especially raised levels of triglycerides, accelerating the cardiovascular complications of diabetes. Genetic factors have been implicated in these complications. The APOE gene is the most studied gene for cardiovascular complications in diabetes. A significant association has been noticed in various countries. Apolipoprotein gene polymorphism is reported as a significant danger for cardiac and vascular complications in subjects with diabetes of different populations and also an important link with hypertension in these subjects [50]. The Apolipoprotein gene polymorphism also affects the plasma lipid profile. There is an association of APOE gene polymorphism with coronary heart disease and stroke but the results are controversial and conflicting [51]. These results are different because of different study designs, and insufficient sample sizes.

Family history and genomic factors are important causes of T2DM and CVD. Lipoprotein-associated factors can be linked with damage to the circulatory system in diabetes. The Apolipoprotein E gene has an effect on the transport of plasma lipoproteins and lipids. It has been shown to be linked with T2DM and ischemic heart disease. A study was carried out by Lebedy et al. in 2016 [48]. In this study, 284 subjects were divided into three groups. Family history, medical history, smoking habits, and history of exercise were asked for through a questionnaire. PCR using restriction enzyme TaqMan^®^ SNP Genotyping Assay was used to assess polymorphism of the Apolipoprotein gene and to see the link of various genotypes with the risk of type 2 diabetes and CVD when the comparison was performed with non-diabetic subjects. It was reported that the E3/E4 genotype of Apolipoprotein was linked with a 2.4 times greater risk of developing CVD (*p* = 0.004). It was also associated with increased levels of total cholesterol and non-HDL-C in all groups. The results are significant and very similar to our observation except that in the present research, there was an 8-fold increase in genotype 4/4 in patients with diabetes and IHD followed by genotype 3/3, 3/4, and 2/3. It is also linked with a significant rise in LDL-C in both T2DM and CVD. However, high-density lipoprotein cholesterol (HDL-C) had no association with APOE polymorphisms in subjects from Tunis. This is different from our results because we found a possible correlation between low levels of HDL and Apolipoprotein gene E polymorphism.

Impaired clearance of lipids by APOE ε4 is due to its higher affinity for LDL-R as compared to other alleles, causing an early “receptor occupation” and accumulation of LDL particles, which suppresses LDL-R synthesis, leading to lower clearance of lipoproteins from the body through LDL-R. A direct relationship between APOE isoforms and premature atherosclerosis has been noticed. The APOE ε4 allele has been linked with the development of both T2DM and CAD [41].

In the present study, the levels of triglycerides, LDL, total cholesterol, and VLDL were raised in the disease group. Levels of HDL were decreased compared to healthy controls. The frequency of the E2/3 genotype was also observed to be higher in diabetics with IHD and stroke. This is consistent with the observations of Kong et al., 2021 [52], in the Chinese population. However, the distribution levels (concentration) of genotypes determined in the present study varied in study groups. From these findings, it could be extracted that genotypes E3/3, E4/4, E2/3, and E 3/4 are responsible for the development of IHD and stroke in type 2 diabetics of Lahore, Pakistan.

## 5. Conclusions

This study, which was conducted in Lahore, Pakistan, found a significant frequency of cardiovascular disorders such as ischemic heart disease (IHD) and stroke among patients with type 2 diabetes, particularly females. Obesity, physical inactivity, poor nutrition, and hypertension, as well as uncontrolled blood sugar levels, were prevalent risk factors among these individuals. Dyslipidemia was also prevalent, with increased cholesterol and triglyceride levels and low HDL. In the present study, frequency of genotype E3/3 was high in disease groups compared to controls. It is present frequently in diabetics with IHD/stroke and patients with both these complications. Genotype 2/3 was most frequently associated with diabetes and stroke or diabetes involving both IHD and stroke. Genotype 3/4 was present frequently in diabetics with stroke. The results demonstrated that genotype 4/4 was present frequently in subjects with diabetes and IHD. It can be concluded that genotypes 3/3, 4/4, 2/3, and 3/4 influence the development of IHD and stroke in type 2 diabetes. These findings point to a hereditary susceptibility for cardiovascular problems in type 2 diabetes.

## Figures and Tables

**Table 1:** Information regarding the study subjects.

S. No.	Variables	Characteristics
1	Total Subjects	260
2	Age	40–85
3	Gender (M/F)	90 (34.6%)/170 (65.3%)
4	Profession	Factory workers, housewives
5	Income	Low income, up to PKR 20,000/month
6	Region	Lahore and its surrounding areas
7	Duration of Onset of Diabetes Range: 2–30 Years	Below 10 years: M: 52 (25%), F: 112 (53.8%) = 78.8% Above 10 years: M: 20 (9.6%), F: 24 (11.5%) = 21.1%
8	Family History of Diabetes	Positive in M: 8 (3.8%), F: 7 (3.3%) = 7.1%
9	Physical Activity (M/F)	M: 15 (7.2%), F: 10 (4.8%) = 12.0%
10	Smoking	30 males (11.5%), one pack of 10 cigarettes/day. No shisha smokers. No female smokers
11	Diet	Oily parathas/bread and curry 2 times a day, deep-fried snacks once a day and less fiber
12	Alcohol	Nil
13	Treatment (Oral/Insulin or Both)	Oral: M: 30 (14.4%), F: 56 (26.9%) = 41.3% Insulin + oral—M: 42 (20.1%), F: 80 (38.4%) = 58.5%
14	Hypertension (Systolic and Diastolic)	Range: (110/75)–(202/110) M: 52 (25.0%), F: 113 (54.2%) = 79.3%
15	BMI < 25 kg/m^2^ BMI > 25 kg/m^2^	Range: 19.6–36 M: 13 (6.2%), F: 2 (0.9%) = 7% M: 59 (28.3%), F: 134 (64.4%) = 92.7%

BMI expressed in kg/m^2^. BP = mmHg. M: Male, F: Female.

**Table 2:** Characteristics of the study groups.

Variables	Groups									
	C		D1		D2		D3		D4	
Gender	M	F	M	F	M	F	M	F	M	F
Subjects	18	34	18	34	18	34	18	34	18	34
Age (years)	48–85	45–85	45–85	47–87	48–75	48–85	61–85	50–67	73–80	54–76
BMI (kg/m^2^)	18.5–22.9	19.5–22.9	30.7–33.7	27.5–32.0	22.9–32.0	21.0–32.0	28.2–32.5	22.1–27.1	26-0–32.0	21.0–28.2
Systolic Blood Pressure (mmHg)	110–120	110–120	135–175	132–202	155–169	140–172	140–175	134–160	144–160	147–165
Diastolic Blood Pressure (mmHg)	75–79	75–79	85–100	85–110	89–100	88–100	85–100	80–100	87–100	85–110

Groups—C: CONTROL; D1: DM Only; D2: DM + IHD; D3: DM + STROKE; D4: DM + STROKE + IHD; BMI = kg/m^2^; B.P = mmHg; M: Male, F: Female.

**Table 3:** Mean ± SEM of the demographic and clinical variables in study groups.

Variables		C	D1	D2	D3	D4	*p*-Value
**Demographics**							
Age (Years)	M	72.40 ± 11.22	64.00 ± 17.81	66.60 ± 16.16	76.40 ± 8.00	77.00 ± 7.88	0.762
	F	65.60 ± 17.42	63.00 ± 17.81	65.40 ± 17.35	59.40 ± 19.4	65.40 ± 14.09	0.762
BMI (kg/m^2^)	M	20.22 ± 0.81	28.04 ± 0.80	26.24 ± 0.99	26.48 ± 0.32	25.22 ± 0.31	0.0001
	F	20.44 ± 0.80	29.10 ± 0.55	30.42 ± 2.03	27.68 ± 0.90	33.36 ± 2.35	0.0001
**Blood Pressure**							
Systolic Blood Pressure (mmHg)	M	114.00 ± 1.70	151.80 ± 6.68	160.20 ± 3.56	152.20 ± 6.71	146.80 ± 5.17	0.0001
	F	112.60 ± 1.32	157.80 ± 11.78	154.20 ± 6.91	143.20 ± 7.27	157.80 ± 3.27	0.0001
Diastolic Blood Pressure (mmHg)	M	77.00 ± 0.70	93.60 ± 2.89	90.60 ± 4.50	93.80 ± 2.85	94.00 ± 2.60	0.0001
	F	77.00 ± 0.70	97.00 ± 4.03	91.00 ± 4.15	91.80 ± 3.55	95.60 ± 1.72	0.0001

Groups—C: CONTROL; D1: DM; D2: DM + IHD; D3: DM + STROKE; D4: DM + STROKE + IHD; BMI = kg/m^2^; B.P = mmHg; M: Male, F: Female.

**Table 4:** Stage of hypertension in the study groups.

Blood Pressure Variation in Disease Group									
Stages of Hypertension	D1		D2		D3		D4		Total % of Patients with Hypertension
	M	F	M	F	M	F	M	F	
HTN Stage 1	1	7	2	8	4	14	6	5	(22.5%) males (6.2%) females (16.3%)
HTN Stage 2	13	22	11	20	9	14	6	23	(56.6%) males (18.7%) females (37.9%)
Normal Blood Pressure	4	5	5	6	5	6	6	6	(20.6%) males (9.6%) females (11.0%)
Total	14	29	13	28	13	28	12	28	(79.3%) males (24.9%) females (54.2%)

Groups—D1: DM ONLY; D2: DM + IHD; D3: DM + STROKE; D4:. DM + IHD + STROKE

**Table 5:** Effect of age on hypertension in study subjects.

Age Group	Hypertension Stage	Percentages (%)	Total % of Subjects with HTN
Age 40–60	Stage 1	10 (4.8%)	(41.8%)
	Stage 2	77 (37.0%)	
	No HTN	6 (2.8%)	
Age 60 or Above	Stage 1 HTN	18 (8.6%)	(37.4%)
	Stage 2 HTN	60 (28.8%)	
	No HTN	37 (17.5%)	

BP = mmHg—Normal: <120/80; Elevated: 120–129/<80; Stage 1: 130–139/80–89; Stage 2: ≥140/≥90.

**Table 6:** Duration of diabetes and stage of hypertension in study subjects.

Duration	No HTN	HTN Stage 1	HTN Stage 2	Total
>10 years	0.8%	4.9%	20.0%	24.9%
<10 years	9.0%	5.4%	49.0%	54.4%

Duration of diabetes >10 years—24.9% of subjects were hypertensive; duration of diabetes <10 years—54.4% of subjects were hypertensive.

**Table 7:** Duration of diabetes and distribution of study subjects.

Duration of Diabetes	Male Percentage	Female Percentage	Total Percentage
>10 Years	20 (9.6%)	24 (11.5%)	(21.1%)
<10 Years	52 (25.0%)	112 (53.8%)	(78.8%)

No. of subjects with >10 years of duration of diabetes—21.1%; no. of subjects with <10 years of duration—78.8%.

**Table 8:** Mean ± SEM of demographic and clinical variables of smokers and non-smokers in study groups.

Variables	C		D1		D2		D3		D4		*p*-Value
	Smoker	Non-Smoker	Smoker	Non-Smoker	Smoker	Non-Smoker	Smoker	Non-Smoker	Smoker	Non-Smoker	
Age (years)	70.00 ± 14.90	71.13 ± 13.80	63.00 ± 18.90	57.20 ± 14.79	70.50 ± 13.50	55.00 ± 13.00	58.00 ± 18.11	67.00 ± 16.65	53.00 ± 13.39	66.25 ± 14.55	0.56
BMI (kg/m^2^)	19.99 ± 0.79	20.00 ± 0.80	28.68 ± 1.62	29.52 ± 0.73	31.85 ± 0.65	26.40 ± 0.70	26.53 ± 2.18	28.76 ± 1.16	28.20 ± 1.05	24.95 ± 1.11	0.05
Systolic Blood Pressure (mmHg)	113.13 ± 1.68	114.90 ± 1.59	155.60 ± 11.63	165.60 ± 9.42	157.00 ± 17.50	140.00 ± 20.00	158.33 ± 12.01	160.00 ± 6.98	142.75 ± 12.87	140.75 ± 9.02	0.05
Diastolic Blood Pressure (mmHg)	76.39 ± 0.69	77.00 ± 0.70	88.20 ± 4.32	91.60 ± 3.98	92.50 ± 2.50	90.00 ± 10.00	91.66 ± 4.41	98.00 ± 4.35	85.50 ± 4.51	89.50 ± 3.88	0.05

Groups—D1: DM ONLY; D2: DM + IHD; D3: DM + STROKE; D4: DM + STROKE + IHD; BMI = kg/m^2^; B.P = mmHg; *p* < 0.05.

**Table 9:** Overall distribution of biochemical parameters in study subjects.

Sr. No.	Variables	Control	Diabetics, mg/dL
1	Fasting Blood Sugar (BSF)	<126 mg/dL	145–356 mg/dL
2	Glycated Hemoglobin (HbA1c)	<5.7%	6.8–13.6%
3	Total Cholesterol (TC)	<150 mg/dL	205–532 mg/dL
4	Total Triglycerides (TGs)	<150 mg/dL	197–433 mg/dL
5	High-Density Lipoproteins (HDLs)	>70–78 mg/dL	36–58 mg/dL
6	Low-Density Lipoproteins (LDLs)	<100 mg/dL	137–255 mg/dL
7	Very-Low-Density Lipoproteins (VLDLs)	2–30 mg/dL	45–90 mg/dL
8	Serum Creatinine	<0.5 mg/dL	0.4–1.1 mg/dL

Diagnostic values: LDL < 100 mg/dL in DM and <70 mg/dL in DM + CHD; HDL (men > 40, women > 50) mg/dL; Triglycerides < 150 mg/dL; Cholesterol < 200 mg/dL in DM and <100 mg/dL in DM + CHD; VLDL, 20–40 mg/dL; HbA1c < 5.7%; BSF > 126 mg/dL; Creatinine < 0.5 mg/dL.

**Table 10:** Distribution of biochemical variables in study groups.

Variables	C		D1		D2		D3		D4	
	M	F	M	F	M	F	M	F	M	F
**Blood Sugar**										
Fasting Blood Sugar mg/dL	73–87	73–83	250–326	260–288	300–339	287–356	166–225	145–220	158–185	148–189
HbA1c %	4.3–5.0	3.9–5.0	6.8–8.8	9.6–13.6	6.9–8.1	7.8–9.5	6.8–8.9	7.3–8.9	7.1–8.4	7.6–8.4
**Lipid Profile**										
TC mg/dL	150–170	160–175	250–277	205–239	200–250	210–231	200–250	217–245	480–532	214–265
TG mg/dL	90–100	90–100	410–433	267–277	220–270	247–285	210–214	300–379	197–220	220–340
HDL mg/dL	70–78	70–78	52–58	40–45	50–55	37–45	44–46	36–40	36–40	41–50
LDL mg/dL	80–90	85–90	132–148	148–165	176–189	184–198	167–189	175–178	238 –255	179–230
VLDL mg/dL	20–30	25–35	80–86	49–55	50–57	47–58	49–55	65–75	80–90	80–85
**Others**										
Serum Creatinine mg/dL	0.7–1.0	0.5–1.0	0.5–1.1	0.5–1.1	0.7–1.0	0.5–1.0	0.7–1.0	0.5–1.0	0.8–1.0	0.7–1.1

Groups—D1: DM ONLY; D2: DM + IHD; D3: DM + STROKE; D4: DM + STROKE + IHD. LDL < 100 mg/dL in DM and <70 mg/dL in DM + CHD; HDL (men > 40, women > 50) mg/dL; Triglycerides < 150 mg/dL; Cholesterol < 200 mg/dL in DM + CHD; VLDL, 20–40 mg/dL; HbA1c < 5.7%; BSF > 126 mg/dL; Creatinine, 0.512 mg/dL.

**Table 11:** Mean ± SEM of biochemical variables in study groups.

Variables		C	D1	D2	D3	D4	*p*-Value
**Blood Sugar**							
BSF	M	79.60 ± 2.63	291.00 ± 12.23	313.80 ± 7.31	187.20 ± 16.68	174.20 ± 5.91	0.0001
	F	81.40 ± 2.80	281.00 ± 7.29	326.60 ± 12.48	184.20 ± 14.75	166.00 ± 7.59	0.0001
HbA1c	M	4.40 ± 0.23	7.62 ± 0.40	7.58 ± 0.26	7.74 ± 0.35	7.32 ± 0.38	0.0001
	F	4.48 ± 0.22	11.50 ± 0.69	8.16 ± 0.63	7.78 ± 0.44	7.74 ± 0.39	0.0001
**Lipid Profile**							
TC	M	120.00 ± 5.00	263.500 ± 13.500	225.000 ± 25.000	225.000 ± 25.000	506.00 ± 26.00	0.0001
	F	127.50 ± 2.50	222.00 ± 17.00	220.50 ± 10.50	231.00 ± 14.00	239.50 ± 25.50	0.0001
TG	M	100.00 ± 0.00	421.50 ± 11.50	245.00 ± 25.00	212.00 ± 2.00	208.50 ± 11.50	0.0001
	F	90.00 ± 0.00	272.00 ± 5.00	266.00 ± 19.00	339.50 ± 39.50	280.00 ± 60.00	0.0001
HDL	M	74.40 ± 4.00	55.00 ± 3.00	52.50 ± 2.50	45.00 ± 1.00	38.00 ± 2.00	0.0001
	F	74.00 ± 4.00	42.50 ± 2.50	41.00 ± 4.00	38.00 ± 2.00	45.50 ± 4.50	0.0001
LDL	M	82.50 ± 7.50	137.00 ± 5.00	182.50 ± 6.50	178.00 ± 11.00	246.50 ± 8.50	0.0001
	F	85.00 ± 5.00	156.50 ± 8.50	191.00 ± 7.00	176.50 ± 1.50	204.50 ± 25.50	0.0001
VLDL	M	25.00 ± 5.00	80.50 ± 35.50	50.00 ± 13.00	48.50 ± 5.50	82.50 ± 0.50	0.0001
	F	31.50 ± 3.50	52.00 ± 3.00	51.50 ± 5.50	70.50 ± 4.50	82.50 ± 2.50	0.0001
**Others**							
Creatinine	M	0.83 ± 0.05	0.86 ± 0.08	0.83 ± 0.05	0.83 ± 0.05	0.89 ± 0.03	0.1614
	F	0.72 ± 0.08	0.90 ± 0.07	0.72 ± 0.08	0.72 ± 0.08	0.90 ± 0.03	0.1614

Groups—C: CONTROL; D1: DM ONLY; D2: DM + IHD; D3: DM + STROKE; D4: DM + STROKE + IHD; M: Male, F: Female.

**Table 12:** Mean ± SEM of biochemical variables of smokers and non-smokers in study groups.

Variables	Groups										
	C		D1		D2		D3		D4		*p*-Value
	Smoker	Non-Smoker	Smoker	Non-Smoker	Smoker	Non-Smoker	Smoker	Non-Smoker	Smoker	Non-Smoker	
BSF	77.62 ± 2.61	76.43 ± 2.59	252.40 ± 34.71	205.60 ± 8.54	186.50 ± 19.50	189.50 ± 6.50	193.00 ± 29.87	173.00 ± 32.45	190.25 ± 11.80	198.75 ± 12.21	0.001
HbA1c	4.52 ± 0.34	4.46 ± 0.31	8.12 ± 0.46	7.90 ± 0.27	7.20 ± 0.10	7.60 ± 1.25	7.83 ± 0.31	7.16 ± 0.61	7.52 ± 0.31	7.42 ± 0.33	0.001
Creatinine	0.83 ± 0.06.	0.80 ± 0.07	0.70 ± 0.07	0.70 ± 0.10	1.00 ± 0.10	0.70 ± 0.10	0.83 ± 0.17	0.70 ± 0.05	0.750 ± 0.06	0.55 ± 0.06	0.10
Total Cholesterol	120.01 ± 4.45	118.23 ± 4.34	216.60 ± 11.50	182.20 ± 21.53	200.50 ± 13.50	180.00 ± 5.00	315.33 ± 108.40	197.00 ± 5.50	210.75 ± 23.03	204.25 ± 14.19	0.001
Triglycerides	99.78 ± 0.01	100.09 ± 0.43	179.00 ± 7.05	176.80 ± 21.59	197.50 ± 0.50	283.50 ± 95.50	166.00 ± 18.50	234.33 ± 52.96	254.25 ± 61.05	206.75 ± 26.94	0.05
HDL	73.45 ± 3.38	73.65 ± 3.32	43.80 ± 3.18	38.00 ± 2.62	45.00 ± 1.00	38.00 ± 2.00	38.33 ± 1.20	43.33 ± 0.66	51.000 ± 9.65	56.50 ± 8.05	0.01
LDL	81.45 ± 7.00	80.91 ± 6.72	161.80 ± 10.78	156.60 ± 18.27	183.00 ± 5.00	159.00 ± 19.00	195.00 ± 30.55	183.00 ± 25.35	143.50 ± 4.368	133.00 ± 15.98	0.001
VLDL	25.02 ± 4.90	25.06 ± 4.89	79.00 ± 30.48	78.39 ± 30.00	50.00 ± 12.98	48.99 ± 13.00	46.49 ± 5.00	47.44 ± 4.48	81.48 ± 2.33	80.43 ± 2.19	0.001

Groups—D1: DM ONLY; D2: DM + IHD; D3: DM + STROKE; D4: DM + STROKE + IHD. LDL < 100 mg/dL in DM and <70 mg/dL in DM + CHD; HDL (men > 40, women > 50) mg/dL; Triglycerides < 150; Cholesterol < 200 mg/dL in DM + CHD; VLDL, 20–40 mg/dL; HbA1c < 5.7%; BSF > 126 mg/dL; Creatinine, 0.5–1.2 mg/dL.

**Table 13:** Genotype distribution of Apolipoprotein E gene in study groups.

Genotype	C	D1	D2	D3	D4	*p*-Value
E3/3	2 (3.8%)	10 (19.2%)	14 (26.9%)	12 (23.0%)	14 (26.9%)	0.0001
E4/4	2 (3.8%)	7 (13.4%)	16 (30.7%)	10 (19.2%)	13 (25%)	0.0001
E2/3	3 (5.7%)	6 (11.5%)	8 (15.3%)	12 (23%)	10 (19.2%)	0.0001
E3/4	3 (5.7%)	8 (15.3%)	11 (21.1%)	13 (25%)	12 (23.0%)	0.0001

*n* = 52 (each group); C: CONTROL; D1: DM ONLY; D2: DM + IHD; D3: DM + STROKE; D4: DM + STROKE + IHD, where *p* ≤ 0.05.

## Data Availability

All the included in the manuscript.
